# Reproducibility and agreement of radiographic assessment of carpal deformities in foals

**DOI:** 10.3389/fvets.2024.1479790

**Published:** 2024-11-07

**Authors:** Alexandre Charles, Xavier Peeters, Constance Verbrugghe, Maxime Vandersmissen, Laurence Evrard, Valeria Busoni

**Affiliations:** Medical Imaging, Clinical Department of Equids, Faculty of Veterinary Medicine, University of Liège, Liège, Belgium

**Keywords:** horse, carpus, carpal conformation, angular limb deformity, deviation, radiography, Pivot Point

## Abstract

**Introduction:**

The Pivot Point (PP) method is commonly used in the radiographic assessment of carpal deformities in young foals, as the range of deviation may influence treatment choice. The aims of this study were to assess the intra- and interobserver reproducibility of the PP method and subjective radiographic evaluation without line drawing to establish the anatomical site responsible for carpal deviation in foals and to evaluate the agreement between these two techniques.

**Material and methods:**

Anonymized radiographs of foals presented for investigation of carpal deformity or prematurity were retrospectively and independently reviewed by six readers. Readers were first asked to subjectively identify the origin of the deviation and then apply the PP method and calculate the angle of deviation (PP_Angle). A second reading in a different randomized order was performed at least two weeks after the first reading. The carpi with the highest variability in PP_Angle measurements were reviewed in consensus by two other radiologists who did not perform the measurements.

**Results:**

A total of 52 radiographs from 25 foals were selected. Good intraobserver reproducibility was observed for all variables, with no significant differences between the first and second readings by the same reader. Measurement of the angle of deviation using the PP method had a high intraobserver reproducibility (correlation coefficient of 0.93, *p* < 0.05). PP and subjective evaluations revealed strong intraobserver reproducibility for the origin of deviation (Cramer coefficients of 0.4 and 0.5, respectively; *p* < 0.05). There was strong agreement between PP and subjective evaluation for establishing the origin of deviation for all readers (Cramer coefficient 0.41; *p* < 0.05). Conversely, interobserver reproducibility for PP and subjective evaluations was low (Kappa values of 0.26 and 0.20, respectively; *p* < 0.05). Higher variability of PP_Angle was found in limbs with lateral bowing of the distal radius.

**Discussion:**

The results of this study suggest that the PP method can reliably be used by the same reader for follow-up of carpal deformities and that there is no need to draw lines if the only required information is the origin of deviation, while measurements by different readers on the same patient may be misleading.

## Introduction

Carpal deformities represent a prevalent and well-known syndrome in foals, characterized by abnormal alignment and rotation of bone segments constitutive of the carpus (1). These deformities can be present at birth or manifest during the initial months of life (2). The 3 primary reported causes of carpal deformities are ligament hyperlaxity, incomplete ossification of cuboid carpal bones and asymmetric development of the distal radial physis (2). These osteoarticular abnormalities are typically assessed in the dorsal plane with description of valgus or varus and origin of the deviation. Less commonly, malalignment in the sagittal plane, referred to as flexural carpal deformities, along with concurrent joint flexion or extension, is also described (2, 3).

Due to the rapid growth experienced in early life, limb deformities can rapidly develop. The growth plates of the long bones typically close in the first year of life, and cuboid carpal bones are fully developed about 18 months of age (4). Therefore early diagnosis and treatment are crucial for successful management of carpal deformities (3).

Conservative management or surgical treatment are used to correct these growth disorders otherwise leading to permanent limb deformity. Treatment approaches are based on the patient clinical condition but also depend on the degree of the angular limb deformity (2, 3, 5). Carpal deviation in the frontal plane less than 4° is considered normal in neonates (6), while when deviation exceeds 4°, a medical or surgical management should be established rapidly. While ligament hyperlaxity and incomplete ossification can often be managed through physiotherapy and/or application of splints, surgical intervention can be recommended for uneven growth plate development (3, 5). Surgery is recommended if a conservative approach fails and/or when deviation is superior to 10° (2, 7).

Radiography is the method currently used to determine the origin and the degree of deviation and is used to monitor its evolution (2, 5). The PP method is the most commonly used method to determine the anatomical site responsible for deviation and to measure the angle of deviation on dorsopalmar radiographs of the carpus that include radial and metacarpal/tarsal diaphysis (5, 8). It consists in drawing 2 lines bisecting the radius and the third metacarpal bone respectively, with the intersection representing the origin of the deviation and the angle between the 2 lines its amplitude/range.

Nonetheless, while intended to offer greater objectivity than subjective visual assessment, this technique remains imperfect (8). Discrepancies in radiographic projection have the potential to lead to erroneous measurement, a concern that becomes critical when deciding the opportunity of surgical treatment based on the 10° cut-off (2, 7).

Therefore, the objectives of this study were first, to assess the intra- and interobserver reproducibility in the radiographic analysis (measurements and origin) of carpal deviation, both subjectively and using the PP method; then, to investigate the agreement of the PP method with subjective radiographic evaluation without line drawing for identification of the site of origin. It was hypothesized that intraobserver reproducibility of the PP method would be good to excellent, while interobserver reproducibility would be lower, particularly between observers of different experience (either students versus veterinarian or radiologists versus surgeons), and that agreement between subjective evaluation and PP method for identification of the origin of the deviation would be good.

## Materials and methods

### Selection and description of subjects

This was a retrospective, secondary analysis study. Case records from the University of Liège from 2014 to 2022 were searched retrospectively for foals less than 6 months of age presented for investigation of carpal deformity or prematurity. Radiographic examination should include at least one dorsopalmar view of one or both carpi. Post-operative radiographs were excluded. Radiographs of foals with a skeletal ossification grade of 1 or 2 out of 4, according to Adams and Poulos (9), were also excluded.

### Radiographic analysis and data recording

Dorsopalmar radiographs were acquired with a phosphide plate detector (Agfa CR with Musica system, Agfa HealthCare NV) or direct digital detector (Agfa DR 10e C with Musica system, Agfa HealthCare NV). Radiographs of each included carpus were randomized, anonymized, and compiled within a file document on a free DICOM medical image viewer (Horos Project, Geneva, Switzerland). Six independent observers, representing 3 groups of different experience (2 ECVDI-certified veterinary radiologist, 2 ECVS-certified surgeons, and 2 final year veterinary students) assessed the radiographs independently. All radiographs were presented to the reader oriented with the lateral aspect on the right.

**Figure 1:**
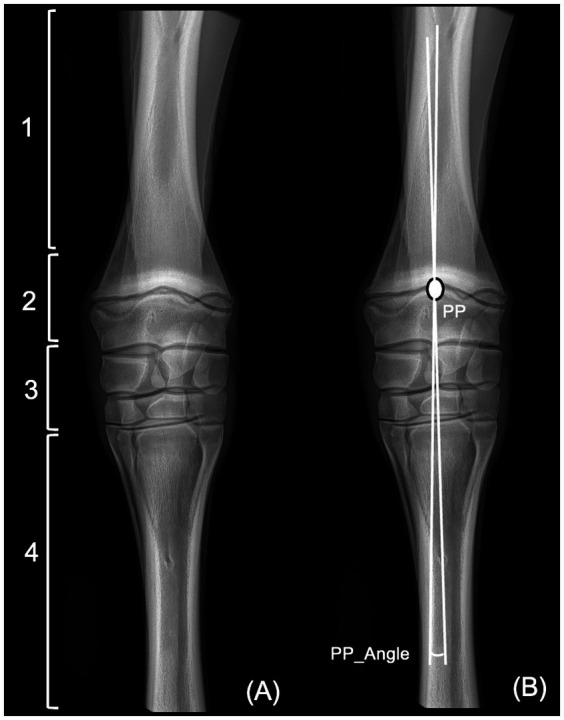
Dorsopalmar radiographic view of the left carpus of a 45 days hanovrian foal. **(A)** Four different categories for the location of the origin of the deviation. 1, radial diaphysis; 2, radial metaphysis or epiphysis; 3, articular; 4, metacarpus. **(B)** Application of the Pivot Point (PP) method. PP denotes the origin of the deviation, corresponding to the intersection of two lines bisecting the radius and the third metacarpal bone, respectively. The angle formed by these two lines is the pivot point angle (PP_Angle).

For each radiograph, observers subjectively assessed image quality, categorizing it as poor, suboptimal or correct (grade 0, 1 or 2 respectively). Subsequently, they were asked to subjectively identify the site origin of the deviation (Orig_dev) as absent (no deviation), located at the radial diaphysis, at the radial metaphysis/epiphysis, articular or in the metacarpal region (Figure 1A). Following this subjective assessment, radiographs underwent geometric evaluation employing the PP method (10). Observers were asked to draw 2 lines bisecting the radius and the third metacarpal bone respectively, measure the angle between the 2 lines and identify their point of intersection (PP), representing the origin of the deviation. The angle formed by these 2 intersecting lines was labeled as the pivot point angle (PP_Angle) (Figure 1B). The PP method was demonstrated to the students on one carpus.

A second radiographic assessment was performed by each observer, at least 2 weeks after the initial reading. Radiographs were presented in a different randomized order.

For each carpus, minimal and maximum angle, mean angle and standard deviation were calculated among all readings. To assess a possible cause of high interobserver discrepancies in PP_Angle, carpi with the highest variability in PP_Angle measures, demonstrated by a standard deviation of more than 2.5°, were reviewed in consensus by 2 other radiologists (one ECVDI resident and one ECVDI-certified veterinary radiologist), who did not perform the measurements.

### Statistics

The normality of the continuous variable PP_Angle was confirmed with a Shapiro test. For the discrete variables image quality, Orig_dev and PP, Kruskal-Wallis and Cramer tests were used to evaluate intraobserver and interobserver reproducibility and agreement between all readers and groups of readers. A Kappa or Chi-square test was used to weight the different levels of agreement between readers. The continuous variable PP_Angle was evaluated with ANOVA and Pearson correlation tests. Statistical tests were repeated after exclusion of radiographs with highest discrepancies in PP_Angle between readers (standard deviation >2.5°). All analyses were conducted with R files (version 3.6.2, R Core Team 2019) and on Microsoft Excel by a statistician. A *p*-value <0.05 was used as criterion for statistical significance.

## Results

A total of 52 radiographs from 25 foals (including 16 males and 9 females) were selected. Median ages of horses was 25 days (range 1–182 days). Various breeds were included, with warmbloods being the most represented (*n* = 14).

The majority (84% of readings—524/624) of the images were considered to have optimal or sub-optimal quality.

A valgus deformity was identified in 35/52 foals, a varus in 3/52 foals, and the deviation was considered irrelevant (<4°) in the remaining 14 foals, based on the mean angle of all readers. Origin of the deviation was identified in the radial metaphysis/epiphysis for almost half of the readings (44 and 49% for subjective and PP evaluation, respectively).

A good intraobserver reproducibility was observed for all variables with no significant differences between first and second reading of the same reader, confirmed by a very strong association of each discrete variable Orig_dev and PP between both readings (Cramer coefficient 0.5 and 0.4, respectively; *p* < 0.05). For angle measurement, Pearson’s test showed a very strong correlation between the two readings (correlation coefficient of 0.93, *p* < 0.05).

Between both readings, radiologists changed significantly less their opinion about Orig_dev and PP compared with surgeons and veterinary students (Chi-square value of 18 for both parameters; *p* < 0.05). When a change occurred, it was not correlated to radiographic quality (*p* < 0.05).

For all readers, there was a significant association between Orig_dev and PP (Cramer coefficient = 0.41 with *p* < 0.05). Both parameters had a significant effect on PP_Angle (*p* = 0.01): carpi with no deviation or metacarpal origin of the deviation having small angles and carpi with radial diaphyseal, epiphyseal or articular origin of the deviation having higher angle measurements. Exclusion of images considered with poor quality (grade 0) by radiologists did not significantly influence other variables between readers or groups of readers.

Interobserver reproducibility was low with only fair agreement for PP and Orig_dev (Kappa value 0.26 and 0.20, respectively; *p* < 0.05). When considering PP_Angle of all readers, maximal angle differences ranged from 2 to 9,58°. Standard deviation from the mean of the measured angle ranged from 0.7° to 3.5°. Discrete variables image quality, Orig_dev and PP demonstrated a fair agreement between readers (respective kappa coefficient of 0.24, 0.25, and 0.20, *p* < 0.05).

Comparing readers’ group, significant differences were identified only for image quality and PP. In contrary there was no significant difference between groups for the subjective Orig_dev and PP_Angle.

**Figure 2:**
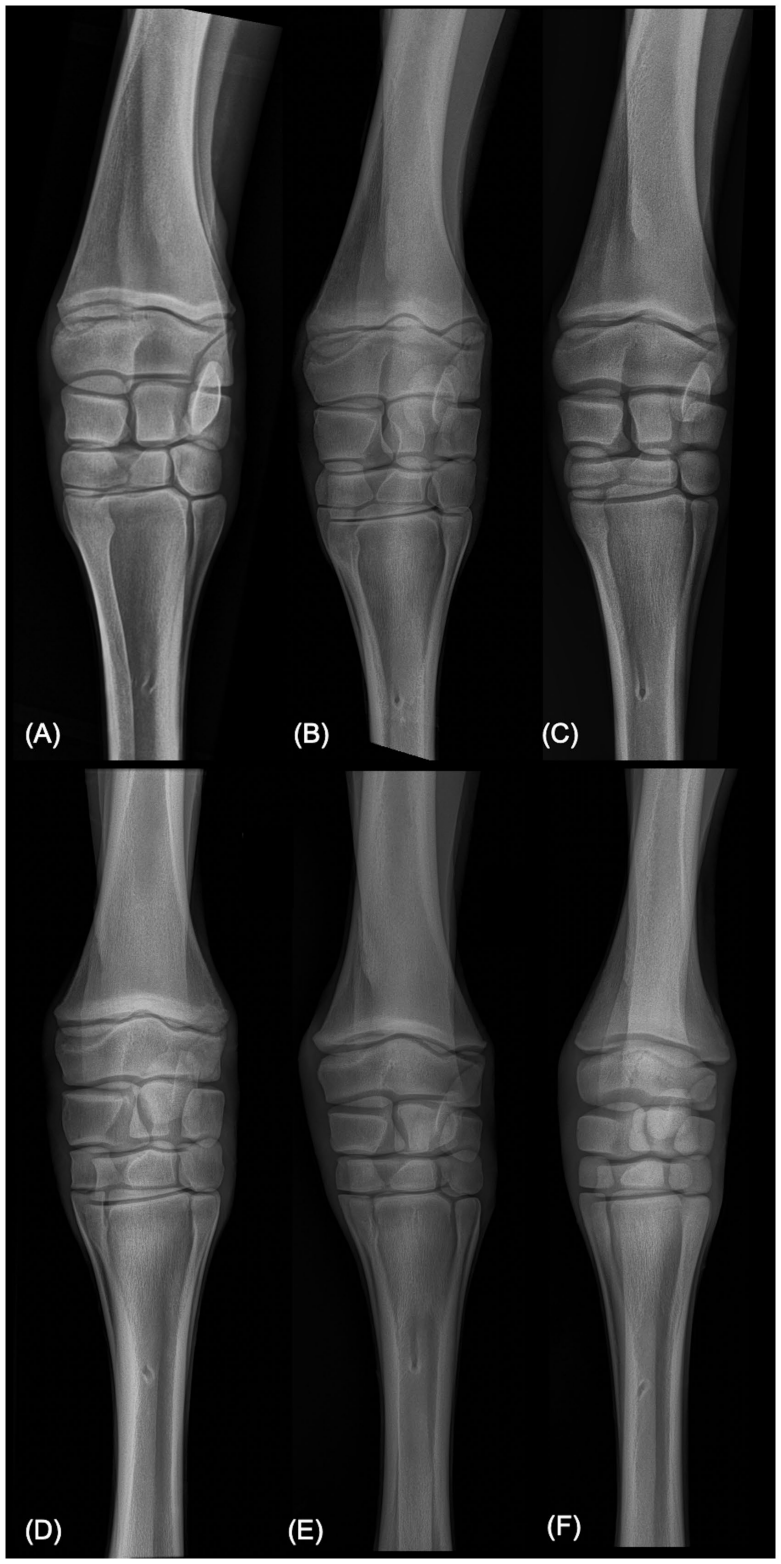
Dorsopalmar radiographic views of carpi of six different foals. **(A–C)** Are carpi with the highest disagreement in PP_Angle between readers. Notice the asymmetric slopes of the medial and lateral radial diaphysis in these three radiographs. **(D–F)** For comparison, are carpi with the highest agreement in PP_Angle between readers.

The highest discrepancies in PP_Angle (standard deviation >2.5°) were found in 5 foals. Reviewing of the radiographs of these foals and their comparison with the others subjects by the non-observers radiologists revealed that all the carpi with high disagreement presented very asymmetric slopes of medial and lateral radial diaphyseal cortices, with a more pronounced concavity of the lateral cortex (Figures 2A–F). When excluded from statistical analysis, no significant differences for PP_Angle was observed between readers.

## Discussion

The PP method is commonly used to assess and quantify carpal deformity in foals (5, 8). The present study aimed to assess intra- and interobserver reproducibility of the PP method and agreement between the PP method and a subjective evaluation to identify the site origin of the deviation.

Intraobserver reproducibility was found to be good. Because there was minimum 2 weeks interval between the readings of the same observer in the present study, good intraobserver reproducibility indicates that the reader has a clear personal understanding of how to draw the lines in the PP method and then applies the same criteria consistently each time. This is consistent with previous studies showing good reproducibility of the PP method and radiographic measurement of carpal parameters (8, 11). Olusa and coauthors showed high intraclass correlation coefficient for all the 14 investigated parameters, including PP_Angle (called medial carpal angle in their study) (11).

Intraobserver reproducibility of radiologists was higher compared to other readers. Experience in radiographic interpretation may explain the more constant opinion of radiologists who have extensive experience in consistently using and interpreting medical images and accurately identifying landmarks for measurements (12, 13).

Interobserver reproducibility was found to be poor. This differs from a previous study demonstrating an excellent correlation between investigators (8). This discrepancy is likely due to that fact that a precise and written guidance about how to trace the lines was not given to the readers in the present study. Therefore, without clear, standardized guidelines, each observer has interpreted the line placement differently, although an “internal standardization” of the technique and the reference points to trace the lines has likely occurred leading to consistency in each reader’s repeated measurements. The article of Brauer and colleagues do not explain how the guidelines were given to the readers so the possibility that the difference interobserver reproducibility due to an imaged and detailed explanation of the PP method given to the readers remains speculative. Visual demonstrations or examples would have likely increased the chances to have a better uniformity in the present study.

The subjective comparison of carpi of higher angle discrepancies with carpi of lower discrepancies suggests that most-likely cause is the inter-individual differences of methods in the placement of the line parallel to the long axis of the radius. This hypothesis is suggested by the fact that all cases with a high disagreement concerned foals with prominent curvature of the lateral cortex of the radius which may have complicated the estimation of the bone axis and/or the bisecting line between the medial and lateral radial cortex. In these cases, readers obtaining the highest measures likely placed the radial line parallel to the medial diaphysis while readers obtaining the lowest angles attempted to place the line in a position bisecting the angle between medial and lateral slopes.

Another technique named individual joint angle method has been proposed for radiographic evaluation of carpal deviations in foals, with excellent interobserver correlation (8). This technique consists in measuring the angle of deviation at the physis and at each carpal joints and then adding these angles to determine the total angle of deviation. Reproducibility of this technique has not been assessed in the present study as the PP method is the most used in equine practice, likely because the individual joint angle technique is more time-consuming. However, we can speculate that because total summated angle is the result of 4 individual joint angles, this method may have reduced interobserver discrepancies in the determination of the total angle of deviation.

Quality of a radiograph is subjective and depends on several factors such as exposure, centering, axis, collimation and patient positioning (4). In the present study it was asked to the reader to grade the “overall quality” of the radiographs, in terms of usability for assessing the carpal deviation, using a three-level semiquantitative scale without any given criteria. Using this overall subjective quality scale, radiographs considered with poor or suboptimal quality had limited influence on other parameters. However, some obliquity of radiographs may potentially have induced an over or under-estimation of the angle of deviation. Limb positioning was not precisely evaluated in the present study. In human medicine, variations of limb position, especially rotation, significantly influence the alignment from standing knee radiographs and standardized radiographs with established landmarks appears essential in recognition of positional error (11, 14). Amount of loading of the limb can also have significant effect on PP_Angle in radiographic assessment of the equine carpal joint of adult horses with increasing of loading lead to progressive increase in PP_Angle (15). Foal positioning is challenging and may have had influence in discrete and continuous variables of the present study.

Why significant differences of several parameters (Orig_Dev & PP_Angle) disappeared when considering groups rather than individual readers remains speculative. Most likely the reason is presence of relatively large differences between the 2 readers of each group and the reduction of individual differences by grouping and averaging of the measures.

The subjective identification of the site of origin of the deviation and the origin determined by the crossing lines (PP) agreed for the same reader in the present study. This is likely due to both methods relying on the same visual markers as landmarks. Since subjective visual markers guide both the subjective judgment and the objective measurement, the consistency in identifying these landmarks ensures that both methods yield similar results for the same reader. Moreover, lines’ placement was performed just after subjective assessment of the origin of the deviation. Therefore, the order of the assessments may have influenced one another with possible increase effect on association between the 2 methods. Even if delayed from subjective assessment, the fact that the initial impression remains present, a cognitive bias named anchoring in daily Medical Imaging practice (16, 17), has likely influenced the PP geometric technique.

Finally, because of the retrospective study design, radiographic measurements were made without any clinical consideration. This is not what happens in authentic clinical practice where visual assessment of the limb deformity on the foal precedes and potentially influences the radiographic interpretation, particularly in surgeons directly dealing with the patient and directly thinking about management and treatment while looking to radiographs.

In conclusion, the results of this study suggest than the PP method can reliably be used by the same reader for follow-up of carpal deformities, while measurements by different readers on the same patient may be misleading. Moreover, the agreement between the subjective assessment and the PP method, suggests that when the measure of the angle is not required, line drawing is not needed to identify the origin of the deviation. Finally high interobserver discrepancy suggests that when the angle is measured for decision making on type of treatment, multiple measurements by different readers, obviously interpreted in conjunction with the clinical assessment of the foal, should be used for final decision to reduce the margin of error.

## Data Availability

The raw data supporting the conclusions of this article will be made available by the authors, without undue reservation. Requests to access these datasets should be directed to alexandre.charles@uliege.be.
